# Cost of illness of non-communicable diseases in private and public health facilities in Nigeria: a qualitative and quantitative approach

**DOI:** 10.11604/pamj.2023.44.6.35494

**Published:** 2023-01-04

**Authors:** Tope Michael Ipinnimo, Olusegun Elijah Elegbede, Kabir Adekunle Durowade, Kayode Rasaq Adewoye, Demilade Olusola Ibirongbe, Paul Oladapo Ajayi, Taofeek Adedayo Sanni, Odunayo Adebukola Temitope Fatunla, Motunrayo Temidayo Ipinnimo, Austine Idowu Ibikunle

**Affiliations:** 1 Department of Community Medicine, Federal Teaching Hospital, Ido-Ekiti, Nigeria,; 2 Department of Community Medicine, Afe Babalola University, Ado Ekiti, Nigeria,; 3 Department of Community Medicine, University of Medical Sciences (UNIMED) Ondo, Nigeria,; 4 Department of Community Medicine, Ekiti State University, Ado-Ekiti, Nigeria,; 5 Department of Paediatrics, College of Medicine & Health Sciences, Afe Babalola University, Ado-Ekiti, Nigeria,; 6 NHIS Unit, Ekiti State University Teaching Hospital, Ado-Ekiti, Nigeria

**Keywords:** Cost of illness, health facilities, Nigeria, non-communicable diseases

## Abstract

**Introduction:**

the cost of illness (COI) of non-communicable diseases (NCDs) has detrimental effects on healthcare outcomes in addition to the serious economic impact on patients and their families. This study estimated and compared the COI of NCDs and its predictors in private and public health facilities (HF) in Ado-Ekiti, Nigeria.

**Methods:**

the study was carried out in selected HF (39 private; 11 public) using a comparative cross-sectional design with a mixed method of data collection. Quantitative data were collected from 348 hypertensive and/or diabetic patients (173 private; 175 public) using a semi-structured, interviewer-administered questionnaire while qualitative data were from 5 key informant interviews (KII) conducted with HF heads or their representatives.

**Results:**

the average monthly COI of NCDs was higher among patients in private (₦15,750.38±14,286.47 [US$43.75±39.68]) than in public HF (₦13,283.37±16,432.68 [US$ 36.90±45.65]) (P<0.001), however, the indirect cost was higher in public HF (private, ₦1,561.07 [US$4.34]; public, ₦3,739.26 [US$10.39]) (p<0.001). Predictors of COI of NCDs identified were income and admission in both groups. Additionally, age, payment method, type of NCDs, having two or more complications, and exercise were identified in private while socioeconomic status, length of diagnosis, and alcohol were identified in public HF. The KII revealed a long waiting time for the public HF patients which accounted for the huge indirect cost.

**Conclusion:**

the study found a huge indirect cost in the public HF that could be minimized by developing policies that would reduce the waiting time of patients. Government and private interventions targeting identified predictors should be applied to reduce the financial burden of NCD.

## Introduction

Non-communicable diseases (NCDs) impose a huge economic burden on countries causing and perpetuating poverty while hindering economic development [1]. They are estimated to cause a global cumulative economic loss of $47 trillion between 2011 and 2030 [1,2]. The economic cost of NCDs from 2015 to 2030 in Costa Rica, Jamaica and Peru was estimated at US$81.96 billion, US$18.45 billion and US$477.33 billion respectively [3]. In Nigeria, the cost of NCDs in the year 2005 was estimated at US$400 million and had risen to about US$8 billion by 2015, a more than twentyfold rise in ten years [4]. The cost of illness, also known as the total cost of managing NCDs is the sum of all the costs components which include direct costs such as medications cost, indirect costs or productivity losses and the intangible cost [5].

The direct and indirect costs of NCDs in public and private health facilities (HF) have a detrimental effect on healthcare outcomes in addition to the serious economic impact on the patients accessing healthcare in these facilities [5,6]. In Bangladesh, the public HF patients pay about US$57.54 for a visit more than the private HF patients, most of which was accounted for by the patients´ income loss [5]. However, some other studies revealed that medical care in public HF was less costly than in private HF [6-8]. In Nigeria, the mean direct cost of chronic illness was estimated at US$137.72 per patient per month and the indirect cost was 24% loss of productivity [9]. Furthermore, the mean monthly direct cost of treating hypertension and diabetes were $104.89 and $159.76 per patient respectively [9].

The cost of illness (COI) could be affected by several factors. In the Netherlands, the main factors increasing healthcare costs were old age and disabling conditions resulting from NCDs [10]. In addition, male gender although being a female patient predicts a higher average direct cost [5], lower socio-economic status/low-income quintile of patients or their household [5,11-13], increased duration of disease [14-17], increased severity of disease [7,11,14-21], and the presence or increased number of co-morbidities were found to increase NCDs COI [16,17]. In Nigeria, male gender, having both hypertension and diabetes as well as an employer paying for care increased the cost of follow-up care by ₦1,132, ₦1,091 and ₦3,735 respectively [22]. COI studies measure the economic burden of disease [23] and translate the adverse effects of diseases into monetary terms, the universal language of decision-makers and the policy arena. Estimates from these studies could be used to provide the basis for policies as well as the economic framework for evaluating health programs and interventions [23,24]. There are presently very few studies on COI of NCDs in Nigeria and none of these studies compares the costs in private and public HF to the best of our knowledge. Previous studies on public HF have demonstrated a high COI [25]. However, little is known about the cost in the private HF, despite being the dominant provider of secondary healthcare in Nigeria [26]. Additionally, these HF differ in their administration and management as well as in their budget and mode of financing. This study, therefore, aims to estimate and compare the COI of NCDs and its predictors in private and public HF in Ado-Ekiti, Nigeria.

## Methods

**Study design and setting:** this was a health facility-based comparative cross-sectional study that used a mixed method of data collection (quantitative and qualitative methods). The study was carried out in Ado-Ekiti, one of the major cities in Southwest, Nigeria. As the state capital of Ekiti State, inhabitants of the city and people from the environs receive healthcare in HF within the city. Ado-Ekiti has a public university teaching hospital, a general hospital, 28 comprehensive, primary and basic health centers, as well as a private university teaching hospital and one hundred and seven other registered private HF. The majority of the population seek healthcare in these HFs while others self-medicate, visit a traditional healer, or seek spiritual healings [27].

**Ethical consideration:** ethical approval for the study was sought and obtained from the Ethics and Research Review Committee of Federal Teaching Hospital, Ido-Ekiti. Written consent for the interview was obtained from all the respondents.

**Participants and study size:** the study included registered adult hypertensive and/or diabetic patients (patients with hypertension only, patients with diabetes only and patients with both diseases) accessing healthcare in private and public HF in Ado-Ekiti, southwest Nigeria. Patients who have accessed healthcare from both public and private HF in the last 3 months were excluded. A minimum sample size of 180 for each group was calculated using the formula for calculating sample size when comparing two means [28]: a 95% confidence interval, 90% power, standard deviation of the COI of NCDs in the general population from a previous study [9], mean COI of NCDs in public [25] and private HF [6] as well as a 10% non-response was assumed. A two-stage sampling technique was used to select eligible respondents. Eleven public and 39 private HF were selected by balloting in stage 1 while systematic random sampling technique was used to select eligible patients in stage 2.

**Variables, data sources/ measurement and bias:** a semi-structured, interviewer-administered questionnaire adapted from previous studies [5,9] was used to collect data from consented eligible patients after seeing the doctor. The questionnaire has 3 sections. Section A elicited socio-demographic and economic characteristics of the patients, section B elicited patients´ health status, characteristics of NCDs and other determinants of the COI and the last section collect data on patients´ monthly healthcare expenditure (direct and indirect cost) on NCDs. Direct costs are expenditures for which payments were made, while indirect costs are those for which resources were lost [24]. Direct costs collected from respondents included the cost of consultation, registration, drugs, consumables, investigations, accommodation, transportation, food as well as other payments made directly during the period of seeking healthcare. The entries were verified from payment receipts and HF records. The indirect cost was calculated using the human capital method, self-reported income or wage loss during the period of illness and healthcare in the HF were estimated [5]. These estimations were done for the patients and their caregivers. The weights and height of respondents were measured using a well-calibrated Omron HN289 digital scale and a portable stadiometer respectively and these were then subsequently used to determine BMI.

**Quantitative data analysis and statistical methods:** data entry and analysis were done with IBM SPSS Statistics for Window, Version 22.0 (IBM Corp., Armonk, N.Y., USA). All entries were checked twice to ensure correctness. Distributions of patients´ characteristics were presented using frequency tables and percentages. Costs were presented as an average with standard deviation in the local currency, Naira (₦) as well as in US dollar (US$), using the Central Bank of Nigeria exchange rate at the mid-point of the data collection year (2019). The chi-square test was used to compare patients´ characteristics between the two groups. Mann-Whitney U test (Wilcoxon 2 sample rank test) was used to compare the COI of NCDs between private and public HF in addition to assessing the association between patients´ characteristics and COI. The correlation coefficient was used to determine the association between COI and continuous variables such as age and income. The multiple linear regression technique was carried out to assess the predictors of COI of NCDs. A P-value of = 0.05 was taken as statistical significance.

**Qualitative data (key informant interview):** five key informant interviews (KII) were conducted with 5 HF administrative heads or their representatives. The five administrative heads or their representatives were purposively selected. Three were selected from private while 2 were from public HF. Data were collected with a KII guide that was designed by the researchers. Information was collected on the COI of NCDs and the factors affecting it. Participants were interviewed on how they charge patients, their experience with clients´ payment patterns and factors affecting the COI of NCDs in their HF. Interviews were conducted after appropriate introduction and consent had been taken from the participants. Interviews were face-to-face and each session lasted about 30 minutes. Notes were taken on paper, voice recording of the discussion with a digital voice recorder was also done. Data from the KII was analyzed using the ATLAS.ti software version 8.0 (this was based on using thematic content analysis). The audio recordings were transcribed verbatim. Analytical framework and codes were developed. The transcripts from the KII sessions were analyzed by focusing on recurrent, dominant and divergent opinions. Themes and sub-themes were generated to address the study objectives and results were presented in prose.

## Results

### Quantitative findings

A total of 360 patients were approached in both private and public HF, of which 348 responded, giving a 96.7% response rate. Of the respondents, 173(49.7%) were in private while 175(50.3%) were in public HF. The mean age ± standard deviation of the respondents was 59.58±10.84 years private, 59.5 ± 10.5 years; public, 59.7±11.2 years) (p=0.910) with the majority of them using paying for their healthcare out-of-pocket (OOP) (private, 90.2%; public, 94.3%) (p=0.152). The number of days of exercise (p < 0.001), level of education (p=0.003) and income (p=0.003) showed significant differences among respondents in the two groups. Details of respondents´ characteristics are in Table 1. Table 2 shows the monthly COI of NCDs among respondents. There was a statistically significant difference in the COI in private and public HF (private, ₦15,750.38 [US$43.75]; public, ₦13,283.37 [US$36.90]) (p < 0.001). The direct COI (private, ₦14,189.31 [US$39.41]; public, ₦9,544.11[US$26.51]) (p<0.001) and the indirect COI (private, ₦1,561.07 [US$4.34]; Public, ₦3,739.26 [US$10.39]) (p < 0.001) also showed statistically significant difference. The indirect COI accounted for less than one-tenth of the COI in private HF but for almost a third in public HF (Private, 9.9%; Public, 28.2%). The factors that were significantly associated with COI of NCDs in private HF were age (r=0.151, p=0.047), payment method (p < 0.001) and in public HF were income (r=0.427, p < 0.001), socioeconomic status (p < 0.001) among others as shown in Table 3. Table 4 shows the result of multiple linear regression analysis of patients´ characteristics and COI of NCDs. In private HF, age, income, payment method, type of NCD, having 2 or more complications, exercise and admission were predictors of COI of NCDs. COI increased by ₦122.96 among respondents in private HF for each additional year in age while holding other variables constant (B=122.96; 95%CI=44.71-201.20; p=0.002). For every additional ₦1.00 rise in the income of private HF respondents, there was a ₦0.05 increase in their COI while holding other variables constant (B=0.05; 95%CI=0.03-0.08; p<0.01). Patients using OOP incurred ₦9,146.24 more than those using national health insurance scheme (NHIS) (B=9146.24; 95%CI=6960.55-11331.93; p < 0.001). The predictors in public HF were income, length of diagnosis, socioeconomic status, alcohol and admission. COI increased by ₦460.37 among respondents in public HF for each additional year in the length of diagnosis while holding other variables constant (B=460.37; 95%CI=77.34-843.41; p=0.019). Those drinking = 14 units of alcohol in a week paid ₦6,577.25 more than respondents drinking <14 units of alcohol in a week (B=6577.25; 95%CI=263.69-12890.81; p=0.041). Respondents with one admission incurred ₦36,353.05 more than those without admission (B=36353.05; 95%CI=29055.51-43650.58; p<0.001). Other characteristics were not found to predict the COI of NCDs in both groups.

**Table 1:** socio-demographic and economic characteristics of respondents

Variables	Health Facility			Test	p-value
	Private (%) n= 173	Public (%) n= 175	Total (%) N=348		
**Mean age±SD(Years)**	59.51±10.49	59.65±11.21	59.58±10.84	-0.113t	0.910
**Sex**					
Male	78 (45.1)	79 (45.1)	157 (45.1)	<0.001x	0.992
Female	95 (54.9)	96 (54.9)	191 (54.9)		
Level of education					
No formal education	7 (4.0)	20 (11.4)	27 (7.8)	13.807x	0.003
Primary education	27 (15.6)	38 (21.7)	65 (18.7)		
Secondary education	52 (30.1)	58 (33.2)	110 (31.5)		
Tertiary education	87 (50.3)	59 (33.7)	146 (42.0)		
**Marital status**					
Unmarried#	52 (30.1)	50 (28.6)	102 (29.3)	0.093x	0.761
Married	121 (69.9)	125 (71.4)	246 (70.7)		
Mean household size±SD	3.75±1.69	3.93±1.77	3.84±1.73	-0.940t	0.348
**Occupation**					
Formalq	59 (34.1)	48 (27.4)	107 (30.7)	2.767x	0.251
Informala	76 (43.9)	92 (52.6)	168 (48.3)		
Retired/Unemployed	38 (22.0)	35 (20.0)	73 (21.0)		
Median income (IQR) (₦)	42,000 (40,000)	35,000 (40,200)	40,000 (41,750)	12326.00m	0.003
**Socioeconomic status**					
Poorest	29 (16.8)	40 (22.9)	69 (19.8)	2.728x	0.604
Poor	35 (20.2)	35 (20.0)	70 (20.2)		
Average	34 (19.7)	35 (20.0)	69 (19.8)		
Rich	36 (20.8)	34 (19.4)	70 (20.1)		
Richest	39 (22.5)	31 (17.7)	70 (20.1)		
**Payment method**					
NHIS	17 (9.8)	10 (5.7)	27 (7.8)	2.056x	0.152
OOP	156 (90.2)	165 (94.3)	321 (92.2)		
**Type of NCD**					
Hypertension only	58 (33.5)	59 (33.6)	117 (33.6)	0.006x	0.997
Diabetes only	57 (33.0)	58 (33.2)	115 (33.1)		
Hypertension and Diabetes	58 (33.5)	58 (33.2)	116 (33.3)		
Median length of diagnosis (IQR)	4.00 (4.50)	4.00 (5.00)	4.00 (5.00)	13722.00m	0.129
**Number of complications**					
0	117 (67.6)	114 (65.1)	231 (66.4)	0.537x	0.764
1	34 (19.7)	40 (22.9)	74 (21.2)		
2 or more	22 (12.7)	21 (12.0)	43 (12.4)		
**Exercise**					
<3 days per week	161 (93.1)	134 (76.6)	295 (84.8)	18.328x	<0.001
≥3 days per week	12 (6.9)	41 (23.4)	53 (15.2)		
**Alcohol**					
<14 units per week	152 (87.9)	151 (86.3)	303 (87.1)	0.192x	0.661
≥14 units per week	21 (12.1)	24 (13.7)	45 (12.9)		
**Smoking**					
Not smoking	157 (90.8)	159 (90.9)	316 (90.8)	0.001x	0.973
Smoking	16 (9.2)	16 (9.1)	32 (9.2)		
**Intake of fruit and vegetable**					
<4 servings per day	148 (85.5)	156 (89.1)	304 (87.4)	1.017x	0.313
≥4servings per day	25 (14.5)	19 (10.9)	44 (12.6)		
**Compliance with salt diet**					
Compliant	113 (65.3)	105 (60.0)	218 (62.6)	1.051x	0.305
Non-compliant	60 (34.7)	70 (40.0)	130 (37.4)		
**Body mass index**					
Normal	50 (28.9)	62 (35.4)	112 (32.2)	1.760x	0.415
Overweight	93 (53.8)	87 (49.7)	180 (51.7)		
Obese	30 (17.3)	26 (14.9)	56 (16.1)		
**Number of clinic visits in the last 1 month**					
0	5 (2.9)	5 (2.9)	10 (2.9)	5.830x	0.054
1	134 (77.4)	116 (66.2)	250 (71.8)		
2 or more	34 (19.7)	54 (30.9)	88 (25.3)		
**Number of admissions in the last 1 month**					
0	155 (89.6)	158 (90.3)	313 (89.9)	0.046x	0.830
1	18 (10.4)	17 (9.7)	35 (10.1)		

x--Chi-square test, t--T-test, m-Mann-Whitney U test, SD—Standard deviation, IQR- Interquartile Range, #--(Unmarried included-Single/Divorced/Widowed), q—(Formal included- Civil servants, Professionals) a—(Informal included Farmers, Traders, Artisans)

**Table 2:** monthly cost of illness of NCDs among respondents

Cost	Health Facility						M-W U	p-value
	Private (n= 173)			Public (n= 175)				
	Average Cost ₦[US$]	D ₦[US$]	Prop. of Total Cost (%)	Average Cost ₦[US$]	SD ₦[US$]	Prop. of Total Cost (%)		
**Registration/Consultation**	2,127.17[5.90]	2,185.05[6.07]	13.51	810.49[2.25]	1,555.75[4.32]	6.10	4377.0	**<0.001**
**Drugs/Consumables**	7,946.71[22.07]	4,391.52[12.20]	50.45	5,109.31[14.19]	4,738.28[13.16]	38.46	7425.0	**<0.001**
**Investigations**	2,313.29[6.43]	2,676.02[7.43]	14.69	2,079.34[5.78]	3,697.29[10.27]	15.65	11413.5	**<0.001**
**Bed/Accommodation**	419.08[1.16]	1,335.71[3.71]	2.66	180.00[0.50]	728.88[2.02]	1.36	14927.5	0.668
**Transportation**	669.19[1.86]	668.46[1.86]	4.25	695.80[1.93]	756.95[2.10]	5.24	14978.5	0.865
**Food**	713.87[1.98]	1,693.31[4.70]	4.53	669.17[1.86]	1,631.55[4.53]	5.04	14605.0	0.558
**Direct cost of illness**	14,189.31[39.41]	11,904.51[33.07]	90.09	9,544.11[26.51]	11,901.36[33.06]	71.85	7832.0	**<0.001**
**Patients Income loss**	972.05[2.70]	1,561.86[4.34]	6.17	2,825.54[7.85]	3,506.65[9.74]	21.27	5038.5	**<0.001**
**Caregivers Income loss**	589.02[1.64]	1,287.82[3.58]	3.74	913.71[2.54]	2,274.68[6.32]	6.88	13241.5	**0.019**
**Indirect cost of illness**	1,561.07[4.34]	2,668.82[7.41]	9.91	3,739.26[10.39]	5,141.95[14.28]	28.15	5872.0	**<0.001**
**Cost of illness**	**15,750.38[43.75]**	**14,286.47[39.68]**	**100.00**	**13,283.37[36.90]**	**16,432.68[45.65]**	**100.00**	**10761.5**	**<0.001**

SD - Standard deviation, M-W U -Mann-Whitney U test.

**Table 3:** socio-demographic, economic and clinical characteristics associated with cost of illness of NCDs

Variable	Health Facility					
	Private (n= 173)			Public (n= 175)		
	Average±SD (₦)	M-WU	p-value	Average±SD (₦)	M-WU	p-value
**Age (years)**		0.151r	0.047		-0.004r	0.963
**Sex**						
Male	16,051.22±15,473.25	3670.50	0.916	14,512.59±15,755.26	3146.00	0.053
Female	15,503.37±13,311.90			12,271.81±16,984.92		
**Level of education**						
No formal education	10,685.71±6,439.15	12.11k	0.007	11,461.90±16,992.29	11.79k	0.008
Primary education	14,313.33±10,908.69			11,821.68±14,048.31		
Secondary education	15,187.98±16,244.53			13,946.46±19,150.86		
Tertiary education	16,940.00±14,439.11			14,232.49±15,052.47		
**Marital status**						
Unmarried#	12,235.00±8,425.72	2707.00	0.146	11,773.88±11,936.52	2788.50	0.266
Married	17,261.12±15,959.93			13,887.16±17,925.62		
**Household size**		-0.002r	0.981		0.081r	0.287
**Occupation**						
Formalq	12,751.10±9,000.28	0.81k	0.666	11,722.08±7,679.44	4.52k	0.104
Informala	15,121.45±11,784.80			13,837.51±19,938.30		
Retired/Unemployed	21,665.00±22,118.34			13,967.94±15,220.49		
**Income (₦)**		0.516r	<0.001		0.427r	**<0.001**
**Socioeconomic status**						
Poorest	9,060.69±2,425.73	45.92k	<0.001	8,185.85±6,489.43	27.76k	**<0.001**
Poor	11,775.71±10,081.99			13,036.80±15,432.73		
Average	14,634.12±15,563.30			14,702.29±20,831.10		
Rich	24,147.08±19,374.87			15,779.03±17,195.41		
Richest	17,514.10±12,355.23			15,800.00±19,334.09		
**Payment method**						
NHIS	3,772.06±1,411.61	21.00	<0.001	11,061.00±6,045.48	725.50	0.522
OOP	17,055.71±14,451.00			13,418.05±16,857.44		
**Type of NCD**						
Hypertension (HTN)	13,565.95±11,391.85	8.29k	0.016	10,818.49±13,833.45	24.73k	**<0.001**
Diabetes (DM)	14,959.12±14,152.58			12,297.21±16,905.07		
HTN and DM	18,712.41±16,580.86			16,776.90±18,003.07		
**Length of diagnosis**		0.209r	0.006		0.249r	0.001
**Number of complications**						
0	10,736.15±4,015.79	32.14k	<0.001	8,137.89±4,242.73	40.85k	**<0.001**
1	23,131.32±18,861.06			18,532.00±24,681.72		
2 or more	31,010.00±23,481.38			31,218.57±22,713.48		
**Exercise**						
<3 days per week	16,236.93±14,678.48	577.00	0.020	14,810.14±18,350.29	2156.00	0.037
≥3 days per week	9,222.50±2,747.39			8,293.41±4,655.62		
**Alcohol**						
<14 units per week	14,535.82±13,302.80	805.00	<0.001	9,709.79±7,968.80	482.50	**<0.001**
≥14 units per week	24,541.43±18,078.92			35,767.08±31,896.38		
**Smoking**						
Not smoking	15,194.94±13,811.86	947.50	0.106	11,084.16±13,051.15	283.00	**<0.001**
Smoking	21,200.63±17,930.05			35,137.94±27,883.38		
**Intake of fruit and vegetable**						
<4 servings per day	16,150.95±14,930.24	1829.00	0.928	13,886.60±17,270.02	1304.00	0.393
≥4servings per day	13,379.00±9,516.06			8,330.53±3,602.12		
**Compliance with salt diet**						
Compliant	14,868.36±13,906.16	2931.50	0.144	10,844.40±9,267.17	3322.50	0.283
Non-compliant	17,411.50±14,953.92			16,941.81±22,997.37		
**Body mass index**						
Normal	11,200.40±4,465.36	3.64k	0.162	9,686.24±6,848.52	6.48k	0.039
Overweight	17,719.41±17,259.23			15,107.79±17,731.27		
Obese	17,229.67±13,470.66			15,756.31±25,167.95		
**Number of clinic visits in the last 1 month**						
0	6,850.00±1,649.24	48.59k	<0.001	10,083.80±6,951.75	31.83k	**<0.001**
1	11,993.69±8,015.36			8,435.60±5,256.15		
2 or more	31,865.00±21,626.15			23,993.33±25,566.80		
**Number of admissions in the last 1 month**						
0	11,315.52±4,542.47	0.001	<0.001	9,229.80±5,781.56	29.00	**<0.001**
1	53,939.44±12,433.99			50,957.65±30,631.52		

SD - Standard deviation, M-W U -Mann-Whitney U test, r-Correlation coefficient, k- Kruskal-Wallis test , #--(Unmarried included-Single/Divorced/Widowed), q—(Formal included- Civil servants, Professionals) a—(Informal included Farmers, Traders, Artisans)

**Table 4:** multiple linear regression relating cost of illness of NCDs to predictor variables

Variable	Health Facility							
	Private (n= 173)				Public (n= 175)			
	B	p-value	95%CI		B	p-value	95%CI	
			Lower	Higher			Lower	Higher
**Age (years)**	122.96	0.002	44.71	201.20	-	-	-	-
**Level of education**								
No formal education R								
Primary education	-835.71	0.648	-4445.99	2774.58	-1292.70	0.662	-7117.72	4532.32
Secondary education	1264.69	0.472	-2197.61	4726.99	-1892.40	0.539	-7969.29	4184.48
Tertiary education	1147.00	0.534	-2490.93	4784.93	-6548.63	0.063	-13465.08	367.83
Income (₦)	0.05	<0.001	0.03	0.08	0.05	0.028	0.01	0.09
**Socioeconomic status**								
Poorest	51.74	0.967	-2448.91	2552.39	-6168.52	0.028	-11673.21	-663.83
Poor	-1733.44	0.086	-3715.77	248.88	-2420.29	0.348	-7503.16	2662.58
**Average R**								
Rich	1252.89	0.254	-908.19	3413.96	1981.29	0.467	-3392.54	7355.11
Richest	938.91	0.529	-2000.32	3878.14	5573.86	0.086	-805.71	11953.42
**Payment method**								
NHIS R								
OOP	9146.24	<0.001	6960.55	11331.93	-	-	-	-
**Type of NCD**								
Hypertension (HTN) R								
Diabetes (DM)	2234.89	0.006	664.78	3805.00	-269.07	0.894	-4246.58	3708.45
HTN and DM	2615.59	0.002	1013.56	4217.62	1871.67	0.360	-2156.78	5900.12
Length of diagnosis	-70.02	0.569	-312.33	172.29	460.37	0.019	77.34	843.41
**Number of complications**								
0 R								
1	450.94	0.628	-1381.36	2283.24	1395.22	0.490	-2587.89	5378.34
2 or more	4842.21	<0.001	2455.57	7228.84	-3206.60	0.307	-9386.37	2973.17
**Exercise**								
<3 days per week	2820.01	0.040	126.31	5513.71	19.10	0.992	-3763.47	3801.67
≥3 days per week R								
**Alcohol**								
<14 units per week R								
≥14 units per week	341.39	0.765	-1910.76	2593.54	6577.25	0.041	263.69	12890.81
**Smoking**								
Not smoking R								
Smoking	-	-	-	-	3283.91	0.345	-3563.16	10130.98
**Body mass index**								
Normal R								
Overweight	-	-	-	-	-1272.25	0.499	-4979.18	2434.68
Obese	-	-	-	-	2543.35	0.320	-2488.67	7575.38
**Number of clinic visits in the last 1 month**								
0 R								
1	1006.16	0.593	-2704.80	4717.11	-3778.64	0.419	-12991.16	5433.87
2 or more	3124.31	0.137	-999.69	7248.32	-1431.58	0.770	-11083.41	8220.25
**Number of admissions in the last 1 month**								
0 R								
1	36559.91	<0.001	34016.81	39103.01	36353.05	<0.001	29055.51	43650.58

B - Beta Coefficient, 95% CI - 95% Confidence Interval, R - Reference Variable, - - Excluded Variable

### Qualitative (Key Informant Interview) findings

The participants included two heads and three representatives of the head of HF. By profession, four are medical doctors while one is a nurse. The main issues discussed were the COI of NCDs and factors affecting it.

### Cost of illness of NCD

Participants from both private and public HF mentioned that the COI of NCDs depends on the type of care received -either in-patient or out-patient care. Also, they agreed that the cost of in-patient care is higher than that of a clinic visit. This can be seen in an extracted excerpt below; *“In a month a patient with any of those diseases will spend an average of ₦5,000 to ₦10,000 for an out-patient visit and for the in-patient care we would be looking at something far more than that, say about ₦30,000 to ₦50,000”*(Representative of the head of a private HF) While speaking on if they consider waiting time and income forgone as part of cost, participants from private HF said patients do not wait for so long in their clinics and thus, not important to consider waiting time and income forgone in calculating the COI of NCDs. However, public HF participants mentioned that their patients wait for a long period, hence waiting time is important and should be considered while calculating COI. They also said that practice of such may be difficult as seen in the subsequent quotation; *“Yes, they should be considered, although it might be difficult in reality. Sometimes patients spend the whole day waiting in the hospital without any compensation at the end, which is not too good”* (Representative of the head of a public HF) On the way they charge their patients, participants in private HF mentioned that charging should be individualized. Private HF individualized their charges depending on what the patients can afford and how much they can pay. This is seen in the extracted excerpts below; *“We have several plans, silver plan, gold plan and platinum plan depending on patients paying ability.”* (Representative of the head of a private HF) Although participants from public HF believed that patient charges should be individualized, however, the participants stated that they do not individualize their charges. A public HF participant is quoted below; *“On a normal day costing is expected to be individualized, however patients in this hospital pay the same amount for most things, for example, the cost of consultation is the same either you are being treated by a consultant, resident or a house officer.”* (Representative of the head of a public HF) All the participants described their experience with clients´ payment after treatment as not forthcoming. They mentioned that, some of the patients owe money and are usually not ready to pay. Many ended up bargaining in order to reduce their bills. This is seen in an extracted excerpt below; *“Majority of patients are not ready to pay for the services they receive and almost all patients bargain including the millionaires even if it is just to give them ₦500.”*(Head of a private HF) The participants from both groups also noted that this clients´ payment pattern is common among patients paying OOP and believe that health insurance can help to prevent it as seen in an extracted excerpt below; *“most patient don´t want to pay out of pocket and this brings the issue of health insurance which is very important because it reduces the burden of payment and out of pocket expenses.”* (Representative of the head of a public HF).

### Factors affecting the cost of illness of NCDs

Participants from both groups mentioned factors that could increase the COI of NCDs as comorbidities and presence of complication(s), need for admission and OOP. In addition, private HF participants mentioned that patients wanting special or private care could increase costs. Some of the participants were quoted saying; *“Somebody that is coming with diabetic foot ulcer will have to pay for the care required for the ulcer in addition to controlling his blood sugar.”*(Representative of the head of a private HF) *“NHIS patients incur less than patients paying outside insurance because they pay only about 10% of their bill”* (Representative of the head of a public HF) Both groups noted that NHIS, a healthy lifestyle and seeking medical intervention early would reduce the COI of NCDs. Also, participants in public HF said that costs will be reduced by time-specific clinic schedules. Some of the participants were quoted saying; *“If people live healthy lifestyle there will be less diagnosis of hypertension and diabetes and even their complications.”*(Head of a private HF) *“We can schedule clinic visits by hours instead of all the patients coming by 7am in the morning including someone that will be seen by 4pm. This long waiting time is adding to the overall cost, so if we can schedule individual patients per time, it would reduce the cost.”*(Representative of the head of a public HF) Participants from public HF mentioned that patients should not have a say in the amount they pay for their care. However, the participants from the private HF believed that although the patients may not have a say in how much they pay, their expectation is what will determine how much to pay as seen in the quote below; *“They cannot determine how much they pay; they can only determine the kind of care they want. Some patients want you to give them the best drug or may want additional tests, this is their expectation and it will affect what they pay.”*(Head of a private HF).

## Discussion

The average monthly COI of NCDs was significantly higher among patients in private (₦15,750.38 [US$43.75]) than in public HF (₦13,283.37 [US$36.90]). A higher COI in private than in public HF has been reported by previous studies [6-8]. These along with the finding in the present study contrast with the finding of a study in Bangladesh where public hospital patients pay more for their healthcare than private hospital patients [5]. The higher COI in private HF in this study could be due to the way private HF are financed in Nigeria. The majority of these HF are owned by individuals who are profit-oriented entrepreneur that sees it as an investment and a source of livelihood. The salaries of staff, running costs and other expenditures by these HF are usually from money generated from the patients unlike in public HF where there is reliance on finances from the state for their expenditures and running. Internally generated revenue from patients in public HF is just an addendum to the funding coming from the Government [29]. The direct COI was significantly higher for private than public HF, and in both makes up a huge proportion of the overall COI. This direct cost is similar to the findings in previous studies in Lagos and Kwara States, Nigeria [30,31]. However, it is lower than what was reported in studies conducted in other parts of the country [9,25,32]. In extremely low income of patients, this huge direct expenditure could make them cut down consumption of basic needs to meet up with payment or even skip appointments as long as they feel well but report back with complications [32] leading to a need for higher healthcare resources.

The indirect COI was significantly higher in public than in private HF. This is similar to the result obtained from a study in Bangladesh [5]. These indirect costs are primarily income losses by patients and their caregivers as a result of accessing healthcare. The higher indirect cost among patients in public HF could be attributed to the long queue and waiting time in these HF. Furthermore, the qualitative aspect of the study buttressed the fact that public HF patients wait longer to access healthcare than private HF patients. This waiting time is however not considered while calculating COI in both groups, even though public HF managers feel it should be considered. It was then suggested that the large indirect COI in public HF could be minimized by reducing the long waiting time through time-specific clinic appointments for patients attending these HF.

In terms of the predictors of COI of NCDs, it was found that COI increases by ₦122.96 for each additional year in the age of patients in private HF. This is consistent with reports from previous studies conducted in different parts of the world [5,11,16,17]. Old-age patients are usually more vulnerable and negatively affected by fees and associated spending on health [5]. Increasing income and higher socioeconomic status were also found to predict higher COI of NCDs. These results contrast some previous literature [5,11-13]. However, socioeconomic status has been found in another study not to be significantly associated with COI [22]. The finding in the present study may be due to the financial capability of the rich and high-income earners to pay for the luxury of care they want and can afford, which the poor may not be able to afford. Evidence from the qualitative aspect of the study shows that client expectation and what they can afford determines how much they pay for their health care. OOP was a positive predictor of higher COI of NCDs in private HF but not in public HF. OOP was also mentioned as a factor that could increase the COI of NCDs in the qualitative aspect of the study and NHIS was seen as a way out of it. This result is consistent with the finding of studies conducted in Nigeria and Bangladesh [5,22]. The lack of significant association between OOP and the COI of NCDs in public HF may be due to the relatively large part contributed by indirect cost to the COI which is not affected by health insurance. NHIS in Nigeria captures the components of the direct COI such as essential drugs, laboratory services and consultations but did not take into consideration the indirect cost [33].

As regards the effect of clinical variables, longer length of diagnosis in public, having both hypertension and diabetes, having two or more complications in private as well as admission in both groups were predictors of higher COI of NCDs. This has further collaborated in the qualitative aspect of the study where comorbidity, presence of complication(s) and need for admission were mentioned as factors that could raise the COI of NCDs. These findings are consistent with that of other studies that found a higher COI among patients with complications [11,14-21,22], patients with longer duration of illness or diagnosis [5,14-17], patients with comorbidity [16,17,22] and those with hospital admission [34]. These clinical variables are a pointer to increase disease severity and a patient with more severe disease will consume more healthcare resources. Studies conducted in different parts of the world have also documented a direct association between higher COI and increase disease severity [11,14,15,17,20]. Modifiable risk factors of NCDs can increase the risk of developing the disease and sometimes the risk of complications and severity. The effect of modifiable risk factors has not been evaluated on the COI of NCDs in previous studies [5,14,34]. However, this study was able to show that NCD patients with controlled risk factors (exercising =3 days/week in private and consuming <14 units/week of alcohol in public) experienced a significant reduction in their COI. This suggests that lifestyle modification will not only reduce the morbidity and mortality due to NCDs but would also reduce the economic burden of the disease.

The strength of this study lies in the mixed data collection method - a qualitative and quantitative approach. This method allows COI to be explored from the professionals´ point of view and not only from that of the researcher. Data were also collected from both the supply and demand side of the healthcare system which gave a more encompassing view of the economic impact of NCDs. However, the study design being cross-sectional could limit the extent to which inferences can be drawn concerning the causal association among variables. Future research could employ a longitudinal study technique and as well look at the burden of intangible costs of NCDs which was not assessed in this study.

## Conclusion

This study has shown that the COI of NCDs was higher in private than in public HF, however, there was a higher indirect cost in public HF due to long waiting time. Predictors of COI of NCDs identified were income and admission in both groups. In addition, age, OOP, type of NCDs, having 2 or more complications and exercise were predictors in private HF while socioeconomic status, length of diagnosis and alcohol were identified in public HF. It is therefore recommended that public HF should employ policies that would minimize the waiting time of patients to reduce the huge indirect cost. Individuals should also engage in lifestyle modifications such as the consumption of a safe level of alcohol and adequate physical exercise. Other identified predictors should be appropriately modified to achieve a reduction of COI due to NCD.

### What is known about this topic

- *There is a huge direct cost of illness of non-communicable diseases in public health facilities in Nigeria, that has continued to rise;*
- *Sociodemographic and economic factors have been found to affect this direct cost of NCDs care*.

### What this study adds

- *This study estimated both the direct and indirect cost of illness of non-communicable diseases in private and public health institutions and found a higher total cost of illness in private health facilities;*
- *However, there was a higher indirect cost in public health facilities and the qualitative aspect revealed that it was due to the long waiting time in these health facilities;*
- *Lastly, this study linked non-communicable diseases risk factors with the economic burden of care and showed that, lifestyle modification would potentially reduce the cost burden*.

## References

[1] The Henry J Kaiser Family foundation. The U.S. Government and Global Non-Communicable Disease Efforts.

[2] Chen S, Kuhn M, Prettner K, Bloom DE (2018). The macroeconomic burden of noncommunicable diseases in the United States: Estimates and projections. PLoS ONE.

[3] Bloom DE, Chen S, Mcgovern ME (2018). The economic burden of noncommunicable diseases and mental health conditions: results for Costa Rica , Jamaica, and Peru. Pan American Journal of Public Health.

[4] Akintunde TS, Akintunde AA, Adeomi AA (2018). Economic burden and psycho-social implications of Non-Communicable Diseases on adults and their households in South-west Nigeria. Ann Health Res.

[5] Pavel MS, Chakrabarty S, Gow J (2016). Cost of illness for outpatients attending public and private hospitals in Bangladesh. International Journal for Equity in Health.

[6] Subramanian S, Gakunga R, Kibachio J, Gathecha G, Edwards P, Ogola E (2018). Cost and affordability of non-communicable disease screening, diagnosis and treatment in Kenya: Patient payments in the private and public sectors. PLoS ONE.

[7] Alouki K, Delisle H, Besançon S, Baldé N, Sidibé-Traoré A, Drabo J (2015). Simple calculator to estimate the medical cost of diabetes in sub-Saharan Africa. World J Diabetes.

[8] Elrayah-Eliadarous H, Yassin K, Eltom M, Abdelrahman S, Wahlstrom R, Ostenson C (2010). Direct costs for care and glycaemic control in patients with type 2 diabetes in Sudan. Exp Clin Endocrinol Diabetes.

[9] Okediji PT, Ojo AO, Ojo AI, Ojo AS, Ojo OE, Abioye-Kuteyi EA (2017). The Economic Impacts of Chronic Illness on Households of Patients in Ile-Ife, South-Western Nigeria. Cureus.

[10] Meerding WJ, Bonneux L, Polder JJ, Koopmanschap MA, Van der Maas PJ (1998). Demographic and epidemiological determinants of healthcare costs in Netherlands: cost of illness study. BMJ.

[11] Mutyambizi C, Pavlova M, Chola L, Hongoro C, Groot W (2018). Cost of diabetes mellitus in Africa: a systematic review of existing literature. Globalization and Health.

[12] Okoronkwo IL, Ekpemiro JN, Okwor EU, Okpala PU, Adeyemo FO (2015). Economic burden and catastrophic cost among people living with type 2 diabetes mellitus attending a tertiary health institution in south-east zone, Nigeria. BMC Res Notes.

[13] Ipingbemi A, Erhun W (2015). Cost implications of treatment of diabetes mellitus in a secondary healthcare facility in Ibadan. Afr J Med Med Sci.

[14] Bahia LR, Araujo DV, Schaan BD, Dib SA, Negrato CA, Leao MPS (2011). The Costs of Type 2 Diabetes Mellitus Outpatient Care in the Brazilian Public Health System. JVAL.

[15] Ulrich S, Holle R, Wacker M, Stark R, Icks A, Thorand B (2016). Cost burden of type 2 diabetes in Germany: results from the population-based KORA studies. BMJ Open.

[16] Chatterjee S, Riewpaiboon A, Piyauthakit P, Riewpaiboon W, Boupaijit K, Panpuwong N (2011). Cost of diabetes and its complications in Thailand: a complete picture of economic burden. Heal Soc Care Community.

[17] Deerochanawong C, Ferrario A (2013). Diabetes management in Thailand: a literature review of the burden, costs, and outcomes. Globalization and Health.

[18] Mata-Cases M, Casajuana M, Franch-Nadal J, Casellas A, Castell C, Vinagre I (2016). Direct medical costs attributable to type 2 diabetes mellitus: a population-based study in Catalonia, Spain. Eur J Health Econ.

[19] Sortsø C, Green A, Jensen PB, Emneus M (2016). Societal costs of diabetes mellitus in Denmark. Diabet. Med.

[20] Chatterjee S, Riewpaiboon A, Piyauthakit P, Riewpaiboon W (2011). Cost of informal care for diabetic patients in Thailand. Prim Care Diabetes.

[21] Suleiman IA, Festus JA (2015). Cost of illness among diabetes mellitus patients in Niger Delta, Nigeria. J Pharm Heal Serv Res.

[22] Bolarinwa O, Abdulahi A, Sanya E, Kolo P, Ameen H, Durowade K (2018). Predictors of Cost of Follow-up Care among Patients with Hypertension and Diabetes Mellitus Attending a Teaching Hospital, North Central, Nigeria. J Heal Sci Res.

[23] Byford S, Torgerson DJ, Raftery J (2000). Cost of illness studies. BMJ.

[24] Rice DP (2000). Cost of Illness Studies: What is Good About Them Injury Prevention?.

[25] Ogaji DS, Nwi-ue LB, Agalah HN, Ibok SG, N-ue DM (2015). Impact and Contributors to Cost of Managing Long Term Conditions in a University Hospital in Nigeria. Journal of Community Medicine and Primary Health Care.

[26] U.S. Department of commerce 2016. Top Markets Report Medical Devices.

[27] Adewoye KR, Aremu SK, Ipinnimo TM, Salawu IA, Orewole TO, Bakare A (2019). Awareness and Practice of Proper Health Seeking Behaviour and Determinant of Self-Medication among Physicians and Nurses in a Tertiary Hospital in Southwest Nigeria. Open Journal of Epidemiology.

[28] Bamgboye AE (2014). Medical Statistics.

[29] Adepoju P A breakdown of Nigeria’s 2020 budget for health.

[30] Bakare OQ, Akinyinka MR, Goodman O, Kuyinu YA, Wright OK, Adeniran A (2016). Antihypertensive use, prescription patterns, and cost of medications in a Teaching Hospital in Lagos, Nigeria. Nigeria Journal of Clinical Practice.

[31] Rosendaal NTA, Hendriks ME, Verhagen MD, Bolarinwa OA, Sanya EO, Kolo PM (2016). Costs and Cost-Effectiveness of Hypertension Screening and Treatment in Adults with Hypertension in Rural Nigeria in the Context of a Health Insurance Program. PLoS ONE.

[32] Okoronkwo IL, Ekpemiro JN, Onwujekwe OE, Nwaneri AC, Iheanacho PN (2016). Socioeconomic Inequities and Payment Coping Mechanisms used in the Treatment of Type 2 Diabetes Mellitus in Nigeria. Niger J Clin Pract.

[33] Okpani AI, Abimbola S (2015). Operationalizing Universal Health Coverage in Nigeria through Social Health Insurance. NMJ.

[34] Diez JDM, Garrido PC, Carballo MG, Miguel AG De, Gutierrez JR, Cano JMB (2008). Determinants and predictors of the cost of COPD in primary care: A Spanish perspective. Int J Chron Obstruct Pulmon Dis.

